# Secretory phospholipase A2-IIa upregulates HER/HER2-elicited signaling in lung cancer cells

**DOI:** 10.3892/ijo.2014.2486

**Published:** 2014-06-10

**Authors:** ZHONGYUN DONG, JAROSLAW MELLER, PAUL SUCCOP, JIANG WANG, KATHRYN WIKENHEISER-BROKAMP, SANDRA STARNES, SHAN LU

**Affiliations:** 1 Department of Medicine, University of Cincinnati College of Medicine, Cincinnati, OH 45237, USA; 2 Department of Pathology, University of Cincinnati College of Medicine, Cincinnati, OH 45237, USA; 3 Department of Environmental Health, University of Cincinnati College of Medicine, Cincinnati, OH 45237, USA; 4 Department of Surgery, University of Cincinnati College of Medicine, Cincinnati, OH 45237, USA

**Keywords:** lung cancer, HER/HER2-elicited signaling pathway, secretory phospholipase A2-IIa, ligand, NF-κB

## Abstract

Lung cancer is the leading cause of cancer death worldwide. There is an urgent need for early diagnostic tools and novel therapies in order to increase lung cancer survival. Secretory phospholipase A2 group IIa (sPLA2-IIa) is involved in inflammation, tumorigenesis and metastasis. We were the first to uncover that cancer cells secrete sPLA2-IIa. sPLA2-IIa is overexpressed in almost all specimens of human lung cancers examined and is significantly elevated in the plasma of lung cancer patients. High levels of plasma sPLA2-IIa are significantly associated with advanced stage and decreased overall cancer survival. In this study, we further showed that elevated HER/HER2-PI3K-Akt-NF-κB signaling contributes to sPLA2-IIa overexpression in lung cancer cells. sPLA2-IIa in turn phosphorylates and activates HER2 and HER3 in a time- and dose-dependent manner in lung cancer cells. The structure and sequence-based docking analysis revealed that sPLA2-IIa β hairpin shares structural similarity with the corresponding EGF hairpin. sPLA2-IIa forms an extensive interface with EGFR and brings the two lobes of EGFR into an active conformation. sPLA2-IIa also enhances the NF-κB promoter activity. Anti-sPLA2-IIa antibody, but not the small molecule sPLA2-IIa inhibitor LY315920, significantly inhibits sPLA2-IIa-induced activation of NF-κB promoter. Our findings support the notion that sPLA2-IIa functions as a ligand for the EGFR family of receptors leading to an elevated HER/HER2-elicited signaling. Plasma sPLA2-IIa can potentially serve as lung cancer biomarker and sPLA2-IIa is a potential therapeutic target against lung cancer.

## Introduction

Lung cancer is the leading cause of cancer death in the USA. It was estimated that there were 226,160 new cases and 160,340 deaths in 2012, contributing to approximately 28% of total cancer deaths (1). Non-small cell lung cancer (NSCLC) is the most common type and accounts for at least 85% of all lung cancer cases. Treatment options for NSCLC depend on the stage of disease and include surgery, radiation and chemotherapy (2–5). Most patients present with advanced or metastatic disease, for which chemotherapy is generally recommended as first line treatment. The first line systematic chemotherapy is typically a platinum doublet. However, efficacy is modest with only minimal improvements in clinical outcomes and therapy is often associated with significant toxicity (5). Erlotinib or gefitinib, a small molecule EGFR (HER1) inhibitor, is a standard second line targeted therapy for NSCLC (6,7). However, EGFR protein mutations account for only around 10–15% of all NSCLC (8). The ultimate cause of treatment failure for cancer is the development of resistance to anticancer drugs (9,10). Lack of effective therapy against lung cancer and metastatic disease underscores the urgency to identify novel targets and develop new lines of anticancer drugs.

The 5-year survival rate with stage 1A non-small cell lung cancer (NSCLC) is as high as 73%, strongly suggesting that early detection can significantly increase cancer survival (11). Low dose computed tomography (LDCT) with reduced radiation exposure is now being used for lung cancer screening and can detect lung cancer at an earlier stage (11). The solitary pulmonary nodules (SPNs) of less than 3 cm in diameter are prevalent and present in 10–20% of baseline lung cancer screening (12,13). Histoplasmosis, an infection due to the *Histoplasma capsulatum* fungus, is an epidemic in the Ohio valley, which leads to a lung nodule rate as high as 61% (14). Screening for lung cancer using LDCT has been shown to decrease lung cancer mortality by 20% in the National Lung Screening Trial (NLST) (15). However, 20–50% of patients screened have solitary pulmonary nodules (SPNs) of less than 3 cm in diameter, among which the lung cancer rate is only 3.6%. SPNs may represent ‘early’ lung cancer, ‘slowly growing’ indolent lung cancer or benign lesions such as granuloma (16). SPNs can be challenging to manage and it is difficult to determine which SPNs are malignant. Currently, there is no single non-invasive, economical and reliable test proven to be effective for early diagnosis of lung cancer.

Ten human secretory phospholipase A2 (sPLA2) isoforms, encoded by the distinct genes, have been identified to date (17,18). These enzymes are distributed in trace amounts in a variety of tissues. Secretory phospholipase A2 group IIa (sPLA2-IIa) is found at high levels in activated inflammatory cells, such as activated macrophages, and some cancers. sPLA2-IIa, an NF-κB target gene (19,20), is a phospholipid hydrolase enzyme that mediates the release of arachidonic acid (AA) and lysophosphatidylcholine, which are the precursors of eicosanoids and platelet-activating factor, respectively (17,21). Eicosanoids are products of both sPLA2-IIa and cyclooxygenase-2 (Cox-2) and exert control over many physiologic and pathologic processes, such as inflammation, immunity, tumorigenesis and metastasis. It was reported that elevated eicosanoids, such as prostaglandins, are involved in the pathogenesis of lung cancer (22).

We were the first to uncover that sPLA2-IIa is overexpressed in almost all specimens of human lung cancers examined and is significantly elevated in the blood of lung cancer patients (23). High levels of plasma sPLA2-IIa with the optimum cutoff value of 2.4 ng/ml predict lung cancer as compared to those patients with benign SPNs and are significantly associated with advanced cancer stage and decreased overall cancer survival. The current study shows that elevated HER/HER2-PI3K-Akt-NF-κB signaling contributes to sPLA2-IIa overexpression in lung cancer cells. sPLA2-IIa can functions as a ligand for the EGFR family of receptors and activates HER/HER2-elicited signaling and the NF-κB promoter. These findings reveal an underlying mechanism of sPLA2-IIa overexpression in lung cancer development and progression.

## Materials and methods

### Reagents

RPMI-1640 medium was purchased from Invitrogen (Gaithersburg, MD). Fetal bovine serum (FBS) was purchased from HyClone Laboratories (Logan, UT). Anti-sPLA2-IIa antibody for western blot analysis and reporter assay was obtained from Cayman Chemical (Ann Arbor, MI). P-HER2 and P-HER3 antibodies were from Cell Signaling Technology (Danvers, MA). HER2 and HER3 antibodies were obtained from Santa Cruz Biotechnology (Santa Cruz, CA). Lapatinib and bortezomib were purchased from Selleck Chemicals LLC (Houston, TX).

### Cell culture

Human alveolar adenocarcinoma cell line A549 and non-small cell adenocarcinoma cell line H1975 were maintained in RPMI-1640 medium supplemented with 10% FBS (complete medium) at 37°C in 5% CO_2_.

### Plasmid

Human sPLA2-IIa cDNA was purchased from Origene Technologies, Inc (Rockville, MD). Both sense and antisense sPLA2-IIa cDNA was subcloned into pCR3.1 vector driven by CMV promoter. The resulting plasmid DNAs, CMV-sPLA2-IIa and CMV-sPLA2-IIa-antisense, were used to generate A549-sPLA2-IIa, H1975-sPLA2-IIa, A549-antisense and H1975-antisense stable lines.

### Western blot analysis

Western blot analysis was performed as previously described (24). Briefly, aliquots of samples with the same amount of protein, determined using the Bradford assay (Bio-Rad, Hercules, CA), were mixed with loading buffer (final concentrations of 62.5 mM Tris-HCl, pH 6.8, 2.3% SDS, 100 mM dithiothreitol and 0.005% bromophenol blue), boiled, fractionated in a SDS-PAGE, and transferred onto a 0.45-μm nitrocellulose membrane (Bio-Rad). The filters were blocked with 2% fat-free milk in PBS, and probed with first antibody in PBS containing 0.1% Tween-20 (PBST) and 1% fat-free milk. The membranes were then washed four times in PBST and incubated with horseradish peroxidase-conjugated secondary antibody (Bio-Rad) in PBST containing 1% fat-free milk. After washing four times in PBST, the membranes were visualized using the ECL Western blotting detection system (Amersham Co., Arlington Height, IL).

### Reporter assay

Cells (10^5^/well) were seeded in 12-well tissue culture plates. The next day, Optifect-mediated transfection was used for the transient transfection assay according to the protocol provided by Invitrogen/Life Technologies, Inc. The cells were then treated for 24 h. Subsequently, the cell extracts were prepared and luciferase activity was assessed in a Berthold Detection System (Titertek-Berthold, Pforzheim, Germany) using a kit (Promega, Madison, WI) following the manufacturer’s instruction. For each assay, cell extract (20 μl) was used and the reaction was started by injection of 50 μl of luciferase substrate. Each reaction was measured for 10 sec in the Luminometer. Luciferase activity was defined as light units/mg protein.

### Computational mapping of sPLA2-IIa-EGFR interaction

ClusPro (cluspro.bu.edu), one of the top performing methods for protein docking, was used in conjunction with structure and sequence-based predictions of protein interaction sites to construct putative models of the EGFR-sPLA2-IIa complex. Approximate normal mode analysis and Elastic Network Models, as implemented in AD-ENM (http://enm.lobos.nih.gov/) and HingeProt (http://bioinfo3d.cs.tau.ac.il/HingeProt/), were used for analysis of conformational changes within sPLA2-IIa. Sppider (sppider.cchmc.org) was used to predict additional protein interaction sites within the sPLA2-IIa hairpin. Multiple resolved crystal structures of EGFR were used, including antagonist bound and free forms of EGFR and EGF-bound form of EGFR dimer.

## Results

### Modeling of sPLA2-IIa-EGFR interaction by protein docking and structural analysis

**Figure 1:**
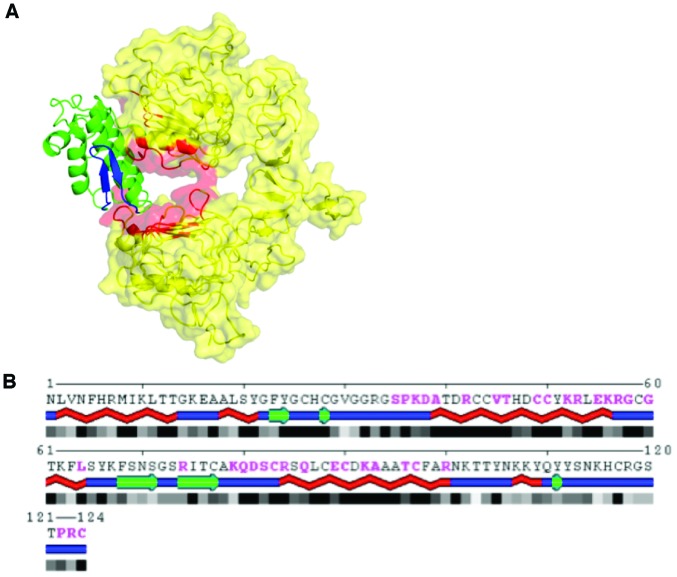
The predicted structure of sPLA2-IIa-EGFR complex. (A) The extracellular domain (ECD) of EGFR in EGF-bound dimer crystal structure conformation is used as the receptor and is shown in yellow, with EGF binding interface highlighted in red. The sPLA2-IIa structure used as the ligand is shown in green, with the putative EGF-like β hairpin within a predicted flexible ‘flap’ domain highlighted in blue. (B) sPLA2-IIa residues predicted to interact with EGF-binding site of EGFR in the top ClusPro model are shown in magenta; helices shown as red braids; β strands as green arrows; and loops as blue lines.

To test the hypothesis that sPLA2-IIa directly interacts with the extracellular domain (ECD) of EGFR in such a way as to stabilize EGFR in its active conformation, we generated putative models of the complex using protein docking and multiple resolved structures of both proteins. Such generated models were assessed using structure and sequence-based predictions of protein interaction sites. In particular, consistency with interaction interfaces predicted in ECD and unbound sPLA2-IIa was used to re-rank docking models. The top predicted model of the sPLA2-IIa-EGFR complex is shown in Fig. 1. The EGFR in EGF-bound dimer crystal structure conformation is used as the receptor and is shown in yellow with the EGF binding interface highlighted in red. The sPLA2-IIa structure used as the ligand is shown in green, with the putative EGF-like β hairpin within a predicted flexible ‘flap’ domain highlighted in blue. This model supports a mode of interaction in which sPLA2-IIa forms an extensive interface with EGFR and brings the two lobes of EGFR into an active conformation, consistenting with EGF bound active form.

While the docking model presented here does not account for the induced fit in sPLA2-IIa, it is worth noting that normal mode analysis of slow coordinated motions, as well as conformational changes between active and inhibited forms of sPLA2-IIa, suggest that the sPLA2-IIa β hairpin shares structural similarity with the corresponding EGF hairpin (blue ‘flap’ in Fig. 1) and could be displaced to provide additional contacts with EGFR. This is further supported by Sppider analysis, which predicts additional protein interaction sites within the sPLA2-IIa hairpin. The information derived from the docking analyses strongly suggests that sPLA2-IIa can directly bind to and extensively interact with the ECD of EGFR, bringing the two lobes of EGFR into an active conformation in a manner analogous with EGF binding to EGFR.

### sPLA2-IIa stimulates the HER/HER2-elicited signaling pathway in a positive feedback manner

**Figure 2:**
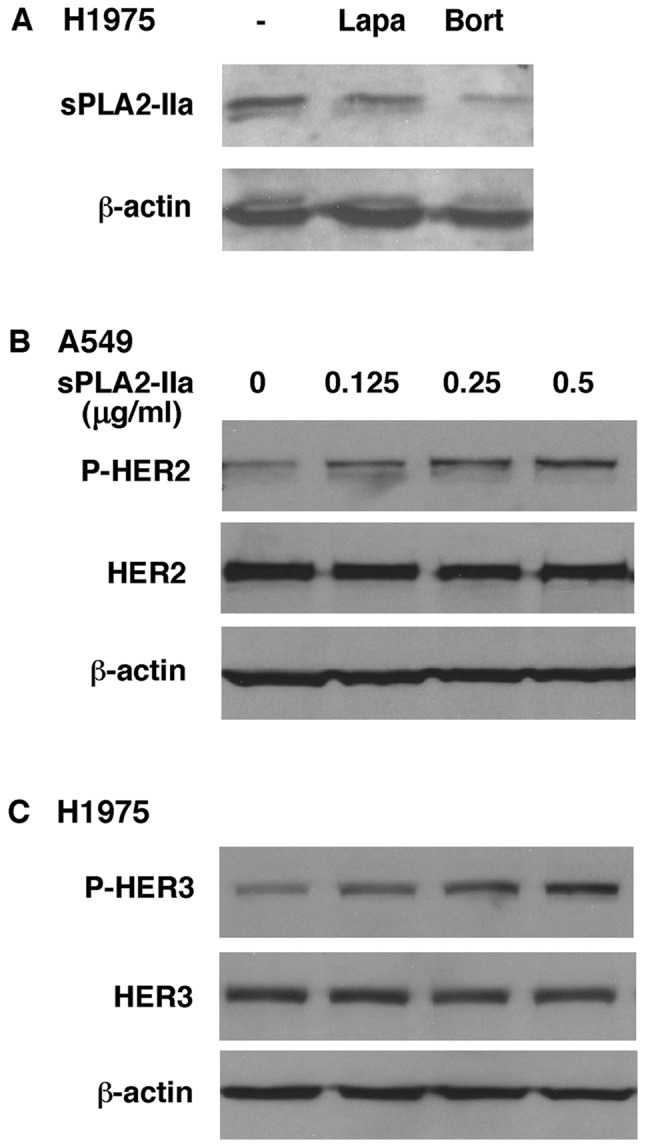
sPLA2-IIa is a potential ligand of the EGFR (HER1) family of receptors. (A) H1975 cells were treated with lapatinib (20 μM) and bortezomib (20 μM) for 24 h. The cell extracts were prepared and subjected to western blot analysis for sPLA2-IIa. (B and C) A549 and H1975 cells were treated with various concentrations of recombinant human sPLA2-IIa for 2 h in the medium containing 10% FBS. The cell extracts were prepared and subjected to western blot analysis for HER2, P-HER2, HER3 and P-HER3.

We examined whether HER/HER2-PI3K-Akt-NF-κB signaling regulates sPLA2-IIa overexpression in lung cancer cells. As we observed in prostate cancer cells (20,25), blocking of HER/HER2 function by lapatinib or NF-κB activity by bortezomib significantly reduces sPLA2-IIa expression in H1975 cells (Fig. 2A). Given that sPLA2-IIa is a secretory protein, there are high levels of sPLA2-IIa in the tumor microenvironment. We determined whether sPLA2-IIa in turn stimulates HER/HER2-elicited signaling in lung cancer cells. Treatment of A549 and H1975 cells with recombinant human sPLA2-IIa phosphorylates and activates HER2 and HER3 in a time- and dose-dependent manner. These data strongly suggest that sPLA2-IIa functions as a ligand (Fig. 1) for the EGFR family of receptors (Fig. 2B and C).

### sPLA2-IIa stimulates the NF-κB promoter activity

**Figure 3:**
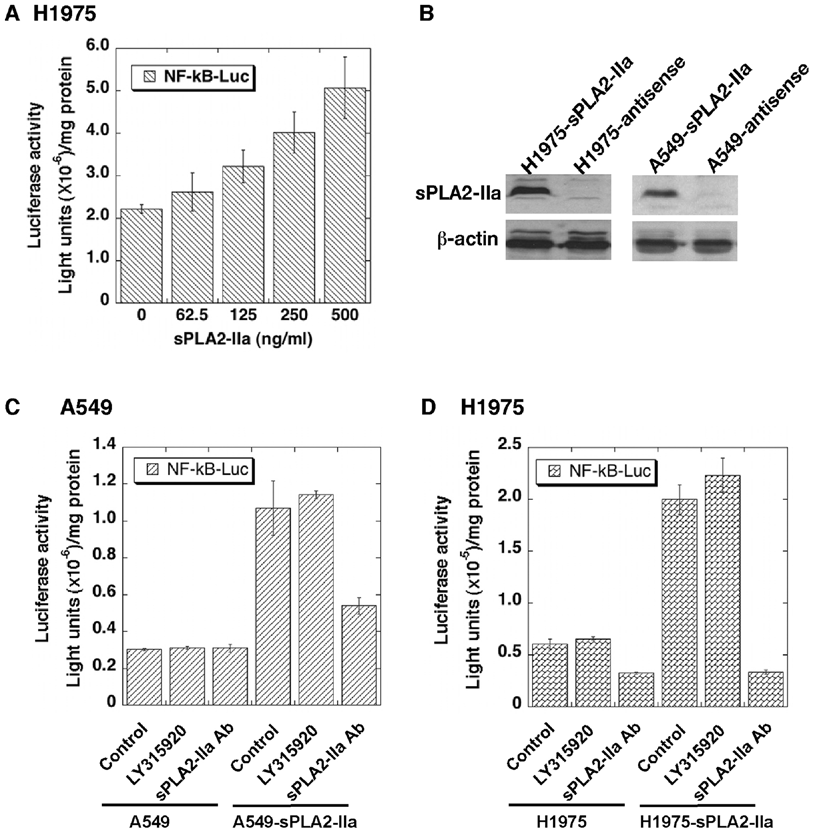
The sPLA2-IIa-induced NF-κB promoter activity is blocked by anti-sPLA2-IIa antibody. (A) H1975 cells (5×10^4^ cells/well) were seeded in a 12-well plate. Next day, the cells were transiently transfected with NF-κB-Luc reporter (0.25 μg/well) overnight. The cells were then treated with various concentrations of recombinant human sPLA2-IIa in the medium containing 10% FBS for 24 h followed by luciferase assay. (B) A549-sPLA2-IIa and H1975-sPLA2-IIa stable line cells overexpress sPLA2-IIa relative to A549-antisense and H1975-antisense stable stable line expressing antisense sPLA2-IIa cDNA, respectively. (C and D) A549-sPLA2-IIA, A549-antisense, H1975-sPLA2-IIa and H1975-antisense stable line cells (5×10^4^ cells/well) were seeded in a 12-well plate. Next day, the cells were transiently transfected with NF-κB-Luc reporter (0.25 μg/well) overnight. The cells were then treated with 200 ng/ml anti-human sPLA2-IIa antibodies or LY315920 (10 μm) for 24 h, followed by luciferase assay.

We determined whether sPLA2-IIa regulates HER/HER2- PI3K-Akt-NF-κB signaling in reporter assays. Recombinant human sPLA2-IIa significantly enhances the NF-κB promoter activity in a dose-dependent manner in H1975 cells (Fig. 3A). We then determined whether endogenous sPLA2-IIa secreted by cancer cells also enhances NF-κB promoter activity. Since A549 and H1975 cells express a low level of sPLA2-IIa, A549-sPLA2-IIa and H1975-sPLA2-IIa stable lines were established and confirmed to overexpress sPLA2-IIa as compared with their control A549-antisense and H1975-antisense stable lines transfected with the antisense sPLA2-IIa cDNA (Fig. 3B). As expected, the basal NF-κB promoter activity is significantly elevated in A549-sPLA2-IIa and H1975-sPLA2-IIa cells relative to A549-antisense and H1975-antisense cells in the reporter assay (Fig. 3C and D). This elevated basal NF-κB promoter activity is inhibited by anti-sPLA2-IIa antibody to block sPLA2-IIa receptor binding, but not by LY315920 to inhibit sPLA2-IIa enzymatic activity. The direct binding of anti-sPLA2-IIa antibody to sPLA2-IIa was validated by the indirect enzyme-linked immunosorbent assay (data not shown).

These data suggest that A549-sPLA2-IIa and H1975-sPLA2-IIa cells overexpress and secrete sPLA2-IIa, and sPLA2-IIa in turn activates the EGFR family of receptors and stimulates HER/HER2-PI3K-Akt-NF-κB signaling (20,25). Anti-sPLA2-IIa antibody blocks sPLA2-IIa ligand function and compromises its activation of the NF-κB promoter activity. This function is not mediated by sPLA2-IIa enzymatic activity, since LY315920 does not alter sPLA2-IIa activity for activation of NF-κB promoter.

## Discussion

Cancer cells overexpress and secret sPLA2-IIa leading to an elevated sPLA2-IIa in the tumor microenvironment and in plasma of lung and prostate cancer patients (20,23,25). Elevated HER/HER2-Vav3-PI3K-Akt-NF-κB signaling induced sPLA2-IIa overexpression and secretion in prostate cancer cells (20,25). However, the underlying role of sPLA2-IIa in cancer development and progression is not known. We found that sPLA2-IIa activates HER/HER2-elicited signaling and the sPLA2-IIa promoter and stimulates androgen-independent tumor cell growth in a positive feedback manner in prostate cancer cells. The current study revealed an underlying molecular mechanism of sPLA2-IIa in cancer development and progression. sPLA2-IIa phosphorylates and activates HER2 and HER3 in a time- and dose-dependent manner in lung cancer cells. sPLA2-IIa also enhances the NF-κB promoter activity. Anti-sPLA2-IIa antibody, but not the small molecule sPLA2-IIa inhibitor LY315920, significantly inhibits sPLA2-IIa-induced activation of the NF-κB promoter. sPLA2-IIa potentially functions as a ligand for the EGFR (HER1) family of receptors and stimulates HER/HER2-PI3K-Akt-NF-κB signaling. The HER/HER2-PI3K-Akt-NF-κB signaling pathway is involved in cancer development, progression, metastasis, and resistance to therapy. Our findings strongly support the notion that plasma sPLA2-IIa can potentially serve as a cancer biomarker and sPLA2-IIa is a potential therapeutic target against cancer.

EGF is a preferable ligand for EGFR/EGFR homodimer or EGFR/HER2 heterodimer, while heregulin-α is a preferable ligand for HER2/HER3 heterodimer (26,27). HER2 has no ligand and HER3 has no tyrosine kinase activity, and they play their function by forming heterodimers with other HER receptors. The HER2/HER3 signaling complex afforded to HER2 by its transphosphorylation of HER3 is highly effective in activating the PI3K/Akt pathway, since HER3, unlike EGFR and HER2, has 6 tyrosine containing binding sites for p85, the regulatory subunit of PI3K. HER3, which signaling function cannot be inhibited by TKIs, provides a focal point in resistance to tyrosine kinase inhibitor (TKI) therapy. An increasing body of evidence highlights the role of HER3 in lung cancer, which has not been addressed in the targeted therapy. ‘One size fit all’ approach is not optimal for lung cancer therapy (28). We found that sPLA2-IIa phosphorylates and activates HER2 and HER3. This finding strongly suggests that sPLA2-IIa overexpression can aberrant stimulating HER/HER2-elicited signaling in lung cancer, contributing to lung cancer development and progression.

The ‘personalized’ treatment will require biomarkers bearing diagnostic and prognostic power to identify the patients who are candidates for the targeted therapy. Clinical efforts have been made to develop new lung cancer therapies targeting HER/HER2-PI3K-Akt-NF-κB signaling using novel inhibitors of this pathway, such as lapatinib and bortezomib (27,29–32). However, there is no convenient and non-invasive lung cancer biomarker test for determining the optimal targeted therapy and monitoring the therapeutic efficacy. We found that high levels of plasma sPLA2-IIa are significantly associated with advanced stage and decreased overall cancer survival (23). Given that sPLA2-IIa is a target gene of HER/HER2-PI3K-Akt-NF-κB signaling, plasma sPLA2-IIa is a potential biomarker for lung cancer.

An increasing body of evidence support the notion that plasma sPLA2-IIa potentially is a novel therapeutic target: i) sPLA2-IIa stimulates tumor cell growth in prostate cancer (20,33,34), colon cancer (35), skin (36), and brain cancer (37,38), while inhibition of eicosanoid signaling leads to cancer regression (39,40); ii) we found that sPLA2-IIa activates HER/HER2-elicited signaling and sPLA2-IIa promoter and stimulates cancer cell growth in a positive feedback manner (20,25,41); iii) sPLA2-IIa stimulates production of vascular endothelial growth factor (VEGF) (42) and nitric oxide synthase expression, contributing to inflammation and angiogenesis in tumors (43,44); iv) sPLA2-IIa abrogates TNF-α-induced apoptosis and compromises immune surveillance function (45); v) sPLA2-IIa stimulates eicosanoid biosynthesis and inflammation contributing to cancer progression (46); vi) through interaction with yet to be identified receptor (18,47), sPLA2-IIa stimulates EGFR-, MAPK-, PI3K/Akt-, NF-κB-mediated cell growth and survival signaling pathways (44,48); and vii) knockdown of secretory phospholipase A2 IIa reduces lung cancer growth both *in vitro* and *in vivo* (49).

In summary, lung cancer cells overexpress and secrete sPLA2-IIa, leading to elevated plasma sPLA2-IIa in lung cancer patients. High levels of plasma sPLA2-IIa are significantly associated with advanced stage and decreased overall cancer survival. Elevated HER/HER2-PI3K-Akt-NF-κB signaling contributes to sPLA2-IIa overexpression in lung cancer cells. sPLA2-IIa potentially functions as a ligand for the EGFR family of receptors and activates HER/HER2-elicited signaling in a positive feedback manner. Anti-sPLA2-IIa antibody significantly inhibits sPLA2-IIa-induced activation of the NF-κB promoter. These findings suggest that plasma sPLA2-IIa can potentially serve as lung cancer biomarker and sPLA2-IIa is potential therapeutic target against lung cancer.
